# Host and geography impact virus diversity in New Zealand’s longfin and shortfin eels

**DOI:** 10.1007/s00705-024-06019-1

**Published:** 2024-03-28

**Authors:** Stephanie J. Waller, Eimear Egan, Shannan Crow, Anthony Charsley, P. Mark Lokman, Erica K. Williams, Edward C. Holmes, Jemma L. Geoghegan

**Affiliations:** 1 Department of Microbiology and Immunology, University of Otago, Dunedin, 9016 New Zealand; 2 National Institute of Water and Atmospheric Research, Auckland, 1010 New Zealand; 3 Department of Zoology, University of Otago, P.O. Box 56, Dunedin, 9054 New Zealand; 4 Sydney Institute for Infectious Diseases, School of Medical Sciences, The University of Sydney, Sydney, NSW 2006 Australia; 5 Institute of Environmental Science and Research, Wellington, New Zealand

## Abstract

The fishing and aquaculture industry is vital for global food security, yet viral diseases can result in mass fish die-off events. Determining the viromes of traditionally understudied species, such as fish, enhances our understanding of the global virosphere and the factors that influence virome composition and disease emergence. Very little is known about the viruses present in New Zealand’s native fish species, including the shortfin eel (*Anguilla australis*) and the longfin eel (*Anguilla dieffenbachii*), both of which are fished culturally by Māori (the indigenous population of New Zealand) and commercially. Through a total RNA metatranscriptomic analysis of longfin and shortfin eels across three different geographic locations in the South Island of New Zealand, we aimed to determine whether viruses had jumped between the two eel species and whether eel virome composition was impacted by life stage, species, and geographic location. We identified nine viral species spanning eight different families, thereby enhancing our understanding of eel virus diversity in New Zealand and the host range of these viral families. Viruses of the family *Flaviviridae* (genus *Hepacivirus*) were widespread and found in both longfin and shortfin eels, indicative of cross-species transmission or virus-host co-divergence. Notably, both host specificity and geographic location appeared to influence eel virome composition, highlighting the complex interaction between viruses, hosts, and their ecosystems. This study broadens our understanding of viromes in aquatic hosts and highlights the importance of gaining baseline knowledge of fish viral abundance and diversity, particularly in aquatic species that are facing population declines.

**Supplementary Information:**

The online version contains supplementary material available at 10.1007/s00705-024-06019-1.

## Introduction

Fisheries and aquaculture represent an essential global industry, providing a significant source of both food and employment worldwide [1]. In 2019, aquatic foods contributed to 17% of the total animal protein globally, with demand increasing yearly [1]. Viruses can pose a major threat to the fisheries and aquaculture industry, particularly in high-density farmed aquatic animals such as salmonid species [2]. Viruses, including infectious pancreatic necrosis virus, cause up to 100% mortality in young salmonid fry [2, 3]. Nevertheless, as more of the virosphere is being documented, it is evident that the vast majority of viruses do not cause overt disease [4–6]. Consequently, it is important to further explore the viromes of relatively under-sampled species, such as fish, to enhance our understanding of factors that drive virome composition and the emergence of viral disease.

Aotearoa New Zealand is home to two native species of freshwater eel: the longfin eel (*Anguilla dieffenbachii)* and the shortfin eel (*Anguilla australis*). The latter can be subdivided into two subspecies; the Australian *Anguilla australis australis* and the New Zealand *Anguilla australis schmidtii*, although some taxonomists do not support the subspecies classification [7, 8]. Based on fossil records and molecular clock dating analysis, eels within the Anguillidae evolved during the Eocene (~50–55 million years ago [mya]) from a marine anguilliform ancestor, while extant species of these eels are believed to have evolved around ~20 mya [9–11]. Longfin and shortfin eels form a distinct phylogenetic Oceanian group and a sister clade to the Atlantic group, comprising the European eel (*Anguilla anguilla*) and the American eel (*Anguilla rostrata*) [11]. Approximately 50 years ago, a third species of eel, the Australian speckled longfin eel (*Anguilla reinhardtii*), arrived in New Zealand [12, 13]. While observations of this species in New Zealand continue to be sporadic, the Australian speckled longfin eel is now recognized as part of New Zealand’s fauna [13]. The New Zealand longfin eel is one of the largest, slowest growing, and longest-lived freshwater eels in the world, and this species is endemic to New Zealand [14–17]. In comparison, shortfin eels, although native to New Zealand, can also be found in eastern Australia and some Pacific islands [17].

Both eel species have a complex catadromous life cycle in which breeding is believed to occur in only a single spawning season when the eels migrate to the South Pacific and spawn [18–20]. Fertilized eggs hatch and develop into larvae, which drift back towards New Zealand on ocean currents [21]. Once the larvae reach the continental shelf, they transform into glass eels. These transparent fish, around 6 centimetres in length, enter freshwater habitats and become pigmented elvers [21], or young ‘yellow’ eels. This freshwater stage, which represents the longest period of an eel’s life, reflects its feeding phase. Years later, on reaching their adult size, eels go through one final transformation, termed silvering, associated with the initiation of puberty and the occurrence of a range of morphological changes that aid in helping them make the long journey back to the South Pacific Ocean to spawn [21–23].

Longfin and shortfin eels have been commercially fished in New Zealand since the mid-1960s [16, 24]. In addition, longfin and shortfin eels are important customary fish to Māori and support mahinga kai (the customary gathering of food and natural materials, and the places where those resources are gathered) [25, 26]. Eels are also one of New Zealand’s top native freshwater apex predators, contributing significantly to maintaining the overall health of freshwater ecosystems by controlling prey populations such as brown trout [27–29]. Based on the New Zealand Department of Conservations 2017 threat status assignments, longfin eels are classified as ‘At risk - Declining’, while shortfin eels are classified as ‘Not Threatened’ [30]. Consequently, with longfin eel populations already under pressure, infectious diseases pose an additional threat. Such threats will potentially adversely impact fisheries and traditional cultural practices but may also change the structure of New Zealand’s freshwater ecosystems if longfin eel populations continue to decline.

To date, very few viruses have been identified infecting New Zealand freshwater eels. Short-finned eel ranavirus was isolated from a visually healthy shortfin eel imported to Italy from New Zealand in 1999 as part of routine screening of live imported fish [31]. While short-finned eel ranavirus was found to cause significant mortality in northern pike (*Esox lucius*), the virus has had minimal to no impact on other hosts, including juvenile black bullhead catfish (*Ameiurus melas*) and shortfin eels [31–33]. Similarly, eel virus European X as well as a picorna-like virus were identified in seemingly healthy longfin eels in 2004 during an investigation into the global distribution of eel viruses [34]. More recently, in 2023, a virological survey in the Chatham Islands – a remote group of islands about 800 km east of New Zealand – identified flaviviruses, nanghoshaviruses, arenaviruses, and highly divergent tosoviruses in eels [35]. None of the viruses identified thus far in either longfin or shortfin eels have been associated with overt disease. Despite this, viral infections may contribute to worldwide eel population declines associated with decreased spawning. For example, European eels infected with eel virus European X developed anemia and hemorrhaging, dying before completing a mock 5500-km migratory distance during swim tunnel experiments, while uninfected eels completed the distance [36]. Viruses including eel virus European, eel virus European X, and anguillid herpesvirus 1, all of which have been detected in wild and farmed eels globally, can cause severe hemorrhagic disease, resulting in significant mortality, although asymptomatic cases are also common [37, 38].

While the two main eel species in New Zealand have partly overlapping distributions, particularly in coastal streams [15], they can have different habitat preferences at different life stages [14, 39]. Generally, shortfin eels tend to populate lowland waterways, while longfin eels prefer inland lakes and rivers [40, 41]. The apparently recent cross-species transmission of eel tosovirus [35] between the two eel species also suggests that they do interact in the wild [35]. Additionally, following the construction of the Manapōuri Lake Control Structure, located at the junction of the Waiau and Mararoa rivers, an eel trap and transfer program was set up to relocate longfin and shortfin elvers from the Manapōuri Lake Control Structure to Lake Manapōuri and Lake Te Anau [42]. The program aimed to allow longfin and shortfin eels to reach inland lakes and rivers that would otherwise be blocked due to hydroelectricity infrastructure [42]. Consequently, these translocations also provided the opportunity for longfin and shortfin eels to interact. In response to the limited research on eel viruses in New Zealand, we used a total RNA metatranscriptomic approach to describe the viromes of longfin and shortfin eels across three sampling sites in the South Island of New Zealand. In particular, we aimed to investigate whether viral richness and abundance were associated with host phylogenetic effects, reveal if eel life stage influenced virome composition, and identify any evidence of viral host jumping between these species.

## Materials and methods

### Permits and animal ethics

Permits were obtained to undertake sampling of freshwater fish on Public Conservation Land (91654 [Permission to operate an electric-fishing device in Public Conservation Land]; and to allow longfin eels to be collected for research purposes (91655 [Research and Collection Permit]). The Otago Animal Ethics Committee approved the use of shortfin eels (animal use protocol number 20-17).

### Characteristics of sampled lakes

Eels were sampled from three lakes in this study: Lake Te Anau, Mavora Lakes, and Te Waihora/Lake Ellesmere. Lake Te Anau is a glacial lake located 202 m above sea level within Fiordland National Park [43]. Lake Te Anau and Lake Manapōuri together make up 73% of New Zealand’s longfin eel lake habitat, which is protected from commercial fishing [44]. There is a flow control structure located at the outlet of Lake Te Anau, which is regulated as part of the Manapoūri Power Scheme [45]. Due to this barrier, elvers are trapped at the Manapōuri Lake Control Structure and are translocated into Lake Te Anau [45].

The Mavora Lakes consist of North Mavora Lake (10.83 km^2^) and South Mavora Lake (1.23 km^2^), which are connected by 1.5 km of the Mavora River [46]. The Mavora Lakes are located 615 m above sea level [46]. Similar to Lake Te Anau, commercial fishing is not allowed, as this area is considered a conservation area by the Department of Conservation. The Manapoūri Control Structure, located at the junction of the Waiau and Mararoa rivers, contains a vertical slot fish pass to allow fish to migrate across the structure [45]. Additionally, elvers are also translocated manually from the Manapōuri Lake Control Structure into Lake Te Anau and Lake Manapōuri [42, 45].

In contrast to Lake Te Anau and the Mavora Lakes, Te Waihora/Lake Ellesmere is a shallow coastal lake that is often regarded as a brackish bar-type lagoon [47]. Unlike Lake Te Anau and the Mavora Lakes, commercial fishing is allowed. However, declining water quality and loss of macrophytes are becoming an increasing concern for Te Waihora/Lake Ellesmere fisheries and conservationists [48, 49].

### Eel liver and gill sample collection

In February and March 2021, 102 longfin eels were sampled from 22 sampling sites within the Te Anau (79 eels) and Mavora Lakes (23 eels) (in the Waiau and Mararoa catchments respectively) in the South Island of New Zealand. Coarse (12 mm) and fine (4 mm) mesh fyke nets were used to sample lake populations. Rivers on the eastern shoreline of Lake Te Anau were also electric fished using a Kainga EFM 300 backpack electric fishing machine (NIWA Instrument Systems). The machine settings were 200–400 volts pulsed direct current, pulse width ~3 milliseconds, and 60 pulses per second. Captured eels were euthanised with an overdose of AQUI-S, and their livers were extracted. Liver tissue samples were submerged in 1 mL of RNAlater and stored at 4°C until they were sent to the University of Otago, Dunedin, where they were stored at -80°C until total RNA was extracted. It is important to note that, while Te Anau and the Mavora Lakes are located close to each other, there are no waterways that directly connect them.

In March 2021, 14 shortfin eels from Te Waihora/Lake Ellesmere were caught by commercial eelers using fyke nets. The captured eels were stored in 12°C spring water before being transported in aerated tanks to Dunedin, where the fish were transferred to 1-cubic-meter circular tanks with recirculating water at 10 parts per thousand salinity at an indoor ambient temperature (14-18°C). Prior to euthanasia, all 14 shortfin eels were used in a study unrelated to the present research involving a 15-day swim experiment in which seven tanks were set up, each containing one yellow and one silver eel. Yellow and silver eels were differentiated based on colour, head shape, eye size, and gonad size. Following the conclusion of the swim experiment, the eels were euthanised with 0.3 g/L benzocaine. The livers and gills were harvested from all 14 shortfin eels. Tissue samples were submerged in 1 mL of RNAlater and stored at -80°C until total RNA was extracted.

A total of 116 tissue samples were collected during 2021. More information regarding sample locations, species, and the number of individual eels caught at each sampling site is provided in Supplementary Table S1.

### Extraction of total RNA from eel livers and gills

Frozen tissue samples stored in RNAlater were thawed, and approximately 30 mg of the tissue was placed in 15-mL RNase-free round-bottom tubes containing lysis buffer. The samples were homogenised for one minute using a TissueRuptor (QIAGEN). Total RNA was then extracted using an RNeasy Plus Mini Kit (QIAGEN) according to the manufacturer’s protocol with minor alterations. Briefly, two ethanol wash steps were added to remove any residual guanidine contamination. Extracted RNA was quantified using a NanoDrop spectrophotometer. RNA was obtained from 111 of the 116 samples at concentrations suitable for downstream processing. Equal volumes of RNA from 4-13 individuals were pooled into 14 libraries based on the sampling location of longfin eels and the life stage, either yellow or silver, of shortfin eels (Supplementary Table S1).

### RNA sequencing

Extracted RNA was subject to total RNA sequencing. Libraries were prepared using a Stranded Total RNA Prep with Ribo-Zero Plus Kit (Illumina). Paired-end 150-bp sequencing of the RNA libraries was performed on an Illumina NovaSeq 6000 platform, using a single S4 lane.

### Virome assembly and virus identification

Paired reads were trimmed and assembled *de novo* using Trinity v2.11 with the “trimmomatic” flag option and default settings [50]. Sequence similarity searches against a local copy of the NCBI nucleotide (nt) database (2021) and the non-redundant (nr) protein database (2021) using BLASTn and Diamond (BLASTx), respectively, were used to annotate assembled contigs [51]. Contigs were categorised into higher kingdoms using the BLASTn “sskingdoms” flag option. Non-viral blast hits including host contigs with sequence similarity to viral sequences (e.g., endogenous viral elements) were removed from further analysis during manual screening. A maximum expected value of 1 × 10^-10^ was used as a cutoff to filter putative viral contigs. Viral contigs that had previously been identified as viral contaminants from laboratory components were also removed from further analysis [52]. Based on the BLASTn and Diamond results (database accessed June 2023), putative viral contigs were analysed using Geneious Prime 2022.2.2 to find and translate open reading frames (ORFs). A nearly complete flavivirus genome sequence from one library was recovered using this approach. This was then used as a reference to which raw reads from other libraries were compared against to obtain more complete flavivirus genome sequences using Bowtie2 with default settings [53].

### Estimating the abundance of viral sequences

Viral abundances were estimated using the “align and estimate” tool in Trinity [54]. RNA-Seq by Expectation-Maximization (RSEM) [55] was selected as the method of abundance estimation, Bowtie2 [53] was used as the alignment method, and the “prep reference” flag was enabled. To mitigate the impact of contamination due to index-hopping, viral sequences with an expected abundance of less than 0.1% of the highest expected abundance for that virus across other libraries were removed from further analysis. Total viral abundance estimates for viruses from vertebrate hosts (i.e., eels) across viral families and orders were compiled across libraries. Estimated abundances were standardised to the number of paired reads per library.

### Phylogenetic analysis

Partial or complete predicted amino acid sequences of the viral RNA-dependent RNA (RdRp) or LO7 (hexon-like protein) [35] of adomaviruses and the replication-associated protein sequences of circoviruses were aligned with those of representatives of the same viral family or order obtained from NCBI RefSeq as well as the closest BLASTp hit, using MAFFT v7.490 (L-INS-I algorithm) (see Supplementary Table S2 for lengths of sequence alignments) [56]. Poorly aligned regions were removed using trimAL v1.2rev59 with the gap threshold flag set to 0.9 [57]. IQ-TREE v1.6.12 was used to construct a maximum-likelihood phylogenetic tree for each viral species/family/order [58]. The LG amino acid substitution model was selected with 1000 ultra-fast bootstrapping replicates for all phylogenetic trees. Phylogenetic trees were annotated using Figtree v1.4.4 [59]. Only those viruses that appeared to be directly infecting the eels, based on their phylogenetic position on the tree, were analysed. All other invertebrate and aquatic-associated viruses that were closely related to and phylogenetically positioned near previously described fish metagenome viruses or invertebrate viruses were omitted from further analysis.

### Analysis of alpha diversity on virome composition

All statistical analysis plots were created using RStudio v2021.09.2 with the tidyverse ggplot2 package [60]. Viral family abundance estimates were first standardised according to the number of raw reads in each library. Standardised viral family abundance estimates were then normalised across each library, and a heatmap was created.

Using the diversity analysis function, which is part of the vegan package [61], the Simpson index (Gini-Simpson), Richness, and Shannon index were selected as the index methods to measure alpha diversity of viral-family-standardised abundance estimates across eel species and location (Lake Te Anau and Mavora Lakes). Welch's *t*-test was used, assuming normal distribution but unequal variance, to determine whether there was a significant difference (*p* < 0.05) in virome alpha diversity between locations (Lake Te Anau and Mavora Lakes).

To analyse whether location affected the virome composition of longfin eels, a distance matrix of standardised eel-family-level virome abundances was created using the vegedist function from the vegan package with Bray-Curtis dissimilarity as the distance measure [61]. The metaMDS function of the vegan package was used to perform multivariate ordination using non-metric multidimensional scaling (NMDS) on the distance matrix [61]. NMDS data points were plotted and coloured by location using ggplot2. The adonis2 function in the vegan package was used to complete a permutational multivariate analysis of variance (PERMANOVA) to test for statistical significance (*p* < 0.05) of the effect of location on virome beta diversity [61].

Full R code and formatted data used in this study are available on GitHub (see Data availability).

### Viral nomenclature

A virus was arbitrarily considered a member of a new species if it shared <90% amino acid sequence identity with the most conserved region (i.e. RdRp/polymerase, LO7, and replication-associated protein sequences) [62, 63] unless otherwise stated. For putative novel virus sequences, we have provided a proposed virus name (subject to formal verification by the International Committee on Taxonomy of Viruses [ICTV]).

## Results

**Fig. 1:**
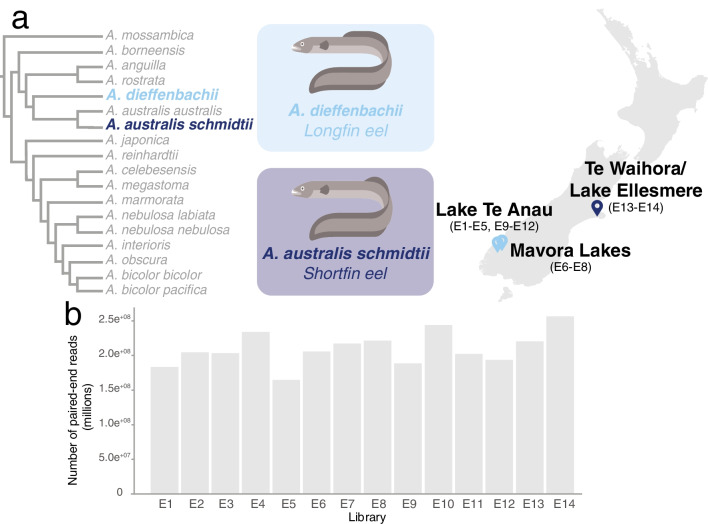
**a** Cladogram (left) illustrating the evolutionary relationships of shortfin eels (*A. australis schmidtii*) and longfin eels (*A. dieffenbachii*) within the family Anguillidae (adapted from Minegishi et al., 2005 [11]). Map of New Zealand (right) indicating the eel sampling locations. Eel illustrations were provided by Hamish Thompson and were used with permission. **b** Total paired-end sequencing reads from eel metatranscriptome libraries

Total RNA from 111 eel samples was pooled into 14 representative samples based on eel species, sample location (of longfin eels), and life stage (of shortfin eels) (see Fig. 1 and Supplementary Table S1). The number of sequencing reads generated from the 14 eel metatranscriptomic libraries varied between 164 and 256 million paired-end reads per library (Fig. 1b).

### Viral abundance and diversity

**Fig. 2:**
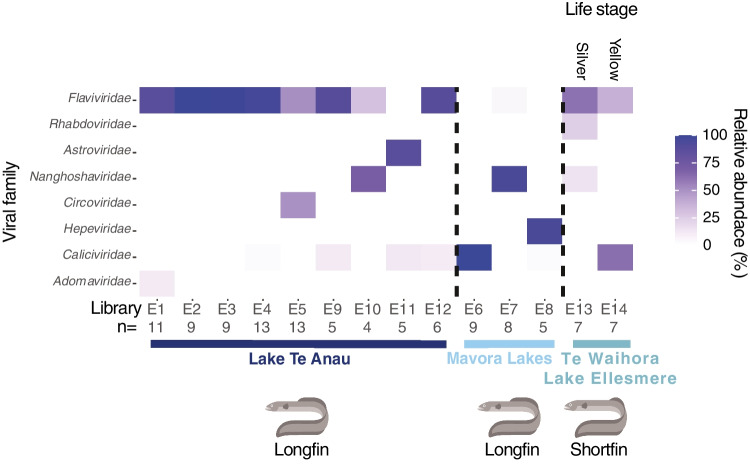
Heatmap of the relative abundance (%) of viruses belonging to different families, normalised by eel library. Information regarding pooled library location, number of individuals within each pool (n), and eel species is provided, as well as the life stage (silver or yellow) of shortfin eels

Analysis of eel metatranscriptomes revealed viral sequences spanning eight different viral families (Fig. 2). Notably, viral sequences from the family *Flaviviridae* (genus *Hepacivirus*) were found in nearly all samples (12 of the 14 libraries). Generally speaking, *Flaviviridae* sequences were highly abundant in longfin eels sampled from Lake Te Anau, with 7 out of the 12 eel libraries having a relative *Flaviviridae* sequence abundance of greater than 50% of the total viral abundance within each library. Besides sequences from the Flaviviridae, viral families were distinct between silver and yellow shortfin eels from Te Waihora/Lake Ellesmere.

### Eel RNA viruses

#### Rhabdoviridae

**Fig. 3:**
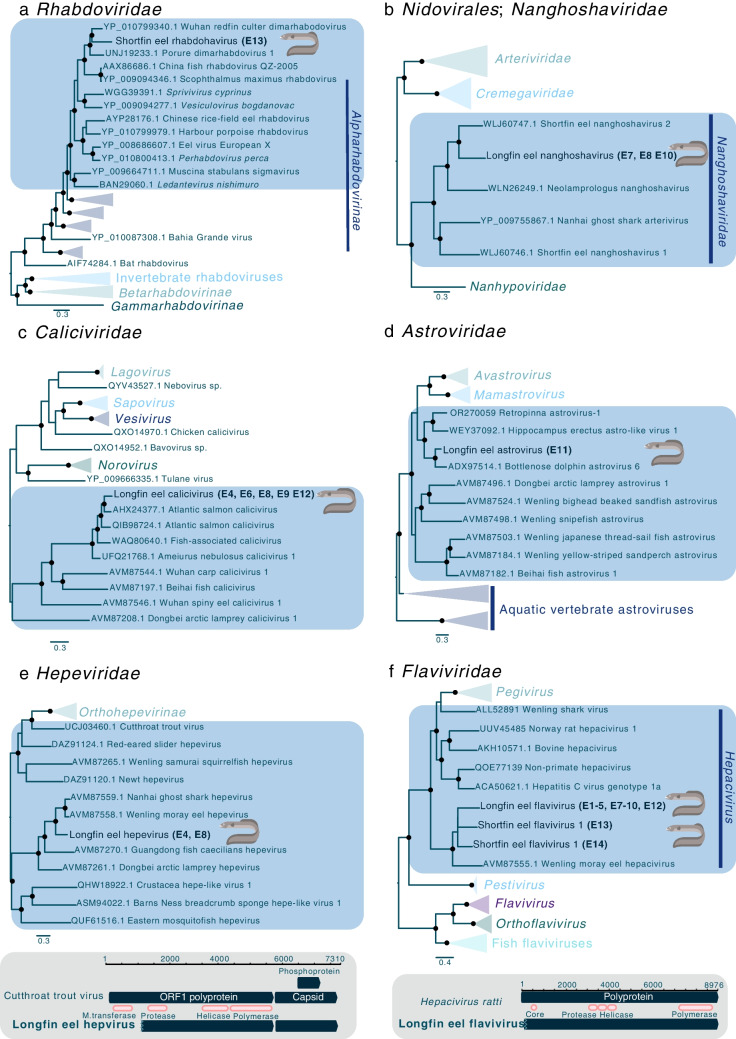
Maximum-likelihood phylogenetic trees of representative viral sequences containing the RdRp from the families (**a**) *Rhabdoviridae*, (**b**) *Nanghoshaviridae* (order *Nidovirales*), (**c**) *Caliciviridae*, (**d**) *Astroviridae,* (**e**) *Hepeviridae*, and (**f**) *Flaviviridae* (genus *Hepacivirus*). The eel viruses identified in this study are shown in bold, and known genera and subfamilies are highlighted. Branches are scaled to the number of amino acid substitutions per site. All phylogenetic trees were rooted at the midpoint. Nodes with ultrafast bootstrap values of >70% are indicated by a black dot. If the near-full-length genome sequence of a virus was determined, its genome organisation is shown below the corresponding phylogenetic tree

Shortfin eel rhabdovirus was identified in a library of shortfin eels from Te Waihora/Lake Ellesmere. The partial sequence of the shortfin eel rhabdovirus L protein, containing the RdRp, shared 56.31% amino acid sequence identity with its closest known genetic relative, Wuhan redfin culter dimarhabodovirus (YP_010799340.1), which was identified previously in a virological survey of healthy predatory carp (*Chanodichthys erythropterus*) from China (Fig. 3a, Supplementary Table S2) [5].

#### Nanghoshaviridae

Longfin eel nanghoshavirus was identified in three libraries of longfin eels from Lake Te Anau and Mavora Lakes (Fig. 3b). The partial longfin eel nanghoshavirus ORF1b protein, containing the RdRp, shared 50.6% amino acid sequence identity with its closest known genetic relative, shortfin eel nanghoshavirus 2, which was identified in seemingly healthy shortfin eels from the Chatham Islands, New Zealand, in 2023 (Supplementary Table S2) [5, 35].

#### Caliciviridae

Five contigs within the family *Caliciviridae* were identified in longfin eel samples from Lake Te Anau and Mavora Lakes. All five contigs shared >90% amino acid sequence identity with each other, suggesting that they likely represent the same viral species, and they were therefore provisionally named longfin eel calicivirus (Fig. 3c). A partial sequence of the longfin eel calicivirus ORF1 polyprotein, containing the RdRp, shared 77.31% amino acid sequence identity with Atlantic salmon calicivirus (AHX24377.1), which was identified previously in Atlantic salmon (*Salmo salar*) (Supplementary Table S2) where it was associated with systemic infection [64, 65].

#### Astroviridae

A single sequence from a member of the family *Astroviridae* was identified in a library of longfin eels sampled from Lake Te Anau (Fig. 3d). This virus was provisionally named longfin eel astrovirus. A partial longfin eel astrovirus ORF1ab polyprotein, containing the RdRp, shared 67% amino acid sequence identity with its closest relative, bottlenose dolphin astrovirus 6 (ADX97514.1), which was identified previously in faeces of a common bottlenose dolphin (*Tursiops truncates*) and is likely associated with fish rather than dolphins (Supplementary Table S2) [66].

#### Hepeviridae

Two sequences comprising a nearly full-length genome sequence of a member of the family *Hepeviridae* were identified in longfin eels from Lake Te Anau and Mavora Lakes. The two partial non-structural polyprotein viral sequences, containing the RdRp, shared >90% amino acid similarity with each other and were provisionally named longfin eel hepevirus (Fig. 3e). The nearly full-length longfin eel hepevirus non-structural polyprotein was most closely related to that of Nanhai ghost shark hepevirus (76.12% amino acid sequence identity, AVM87559.1), while the other partial longfin eel hepevirus non-structural polyprotein shared 65.59% amino acid sequence identity with that of Wenling moray eel hepevirus (65.59% amino acid sequence identity, AVM87558.1), which was identified previously in virological surveys of a healthy species of ghost shark (*Chimaera* sp.) and moray eels (*Gymnothorax reticularis*), respectively (Supplementary Table S2) [5].

#### Flaviviridae

Ten partial flavivirus sequences were found in longfin eels from Te Anau and Mavora Lakes. All 10 partial polyprotein sequences, containing the RdRp, shared >90% sequence identity with each other. This virus, provisionally named longfin eel flavivirus is most closely related to members of the genus *Hepacivirus*. The partial longfin eel flavivirus polyprotein shared 33.32% amino acid sequence identity with that of Wenling moray eel hepacivirus (AVM87555.1), which was identified previously in healthy moray eels (Supplementary Table S2) [5]. A nearly full-length genome sequence (8066 nucleotides [nt]) of this virus was determined (Fig. 3f).

In addition to longfin eel flavivirus, two closely related partial *Flaviviridae* contigs were identified in shortfin eels from Te Waihora/Lake Ellesmere, and this virus was named shortfin eel flavivirus 1. The viruses from these New Zealand eel species formed a monophyletic group (Fig. 3f). Both of these partial flavivirus polyprotein sequences, containing the RdRp, shared ~45% amino acid sequence identity with that of Wenling moray eel hepacivirus. It is important to note, however, that while we have assigned these contigs to the same virus, they did not overlap and therefore could have been derived from different viruses.

### Eel DNA viruses

#### Adomaviridae

**Fig. 4:**
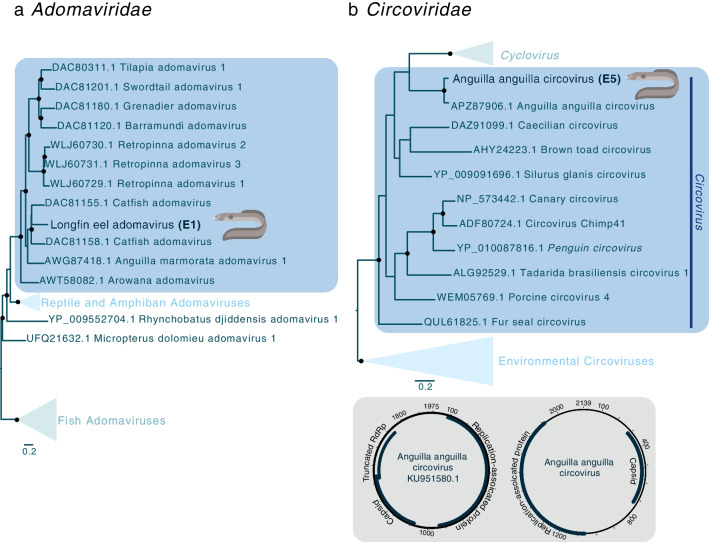
Maximum-likelihood phylogenetic trees of representative viral sequences containing (**a**) the LO7 gene from a member of the family Adomaviridae and (**b**) replication-associated protein gene from a member of the family *Circoviridae*. The eel viruses identified in this study are shown in bold, and known genera and subfamilies are highlighted. Branches are scaled to the number of amino acid substitutions per site. All phylogenetic trees were rooted at the midpoint. Nodes with ultrafast bootstrap values >70% are indicated by a black dot. In the lower panel, the genome organisation of the previously identified Anguilla anguilla circovirus (KU951580.1) and that of the Anguilla anguilla circovirus genome found in longfin eels are shown

A partial longfin eel adomavirus LO7 (hexon-like protein) [35] gene sequence was identified in longfin eels from Lake Te Anau, and the encoded protein was most closely related (56.14% amino acid sequence identity) to that of catfish adomavirus (DAC81155.1) previously identified in a virological survey of healthy yellowhead catfish (*Tachysurus fulvidraco*) (Fig. 4a, Supplementary Table S2) [67].

#### Circoviridae

A full-length circovirus genome was identified in a library of longfin eels from Lake Te Anau (Fig. 4b). The full-length replication-associated protein of this virus shared 91.96% amino acid sequence identity with the replication-associated protein of Anguilla anguilla circovirus (APZ87906.1), which was identified previously in sabre carp (*Pelecus cultratus*) and European eels (*A. anguilla*) from Hungary that showed no signs of disease (Supplementary Table S2) [68]. The full genome of the circovirus found in longfin eels consisted of 2,139 nt, which is similar to the 1,975-nt genome of the previously identified Anguilla anguilla circovirus (KU951580.1). These viruses share 96.28% nt sequence identity, indicating that the same virus infects longfin eels, sabre carp, and European eels. Consequently, we have named this virus "Anguilla anguilla circovirus" (Fig. 4b).

### Factors shaping the diversity of eel viromes

**Fig. 5:**
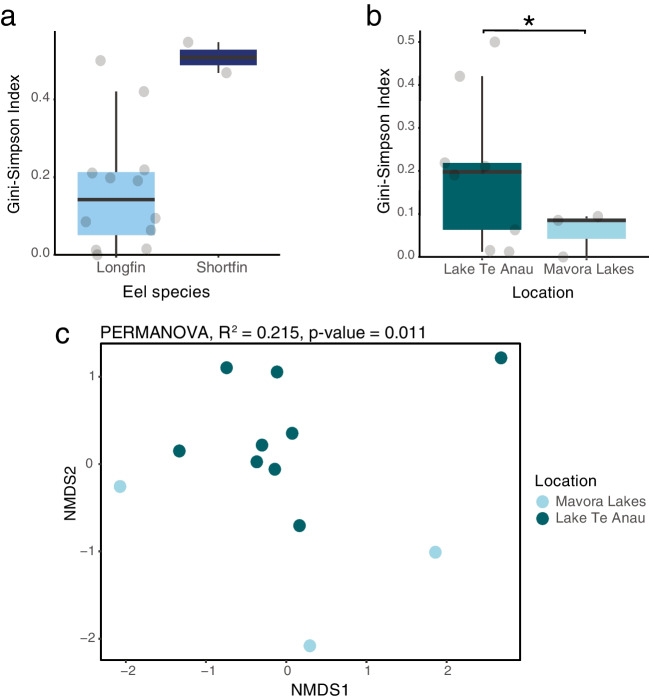
Alpha and beta diversity analysis of eel family-level viromes. (Gini-Simpson Index boxplots of eel viruses across eel libraries in relation to (**a**) eel species (**b**) and location. Significant differences in the Gini-Simpson index, measured using Welch’s *t*-test (*p* < 0.05), are denoted by an asterisk. (**c**) Nonmetric multidimensional scaling (NMDS) plot investigating location on family-level virome composition. The NMDS plot was based on Bray-Curtis dissimilarities and was coloured by location. The effect of location on virome composition of longfin eels was measured using a PERMANOVA

We next investigated whether alpha diversity, measured using the Gini-Simpson index (which accounts for both viral richness and abundance, but weighs more importance on common species), is influenced by host phylogenetic effects (i.e., eel species) or can be better explained by their environment (i.e., sampling location). Shortfin eel viromes appeared to be more diverse than longfin eel viromes, although as there were only two pooled samples of shortfin eels, statistical analysis could not be performed (Fig. 5a). Similarly, Lake Te Anau longfin eel viromes were more diverse, with a mean Gini-Simpson index of 0.2 compared to 0.06 for Mavora longfin eels (Welch’s *t*-test, *p* = 0.048, 95% confidence interval, 0.001- 0.29, degrees of freedom, 9.9706) (Fig. 5b). Additionally, the virome composition of longfin eels from Lake Te Anau and Mavora Lakes were significantly different (permutational multivariate analysis of variance, R^2^ = 0.215; *p* = 0.011) (Fig. 5c). Despite this, there was no significant difference in viral richness (Welch’s *t*-test, *p* = 0.91, 95% confidence interval, -3.546401 - 3.768623, degrees of freedom = 2.1122) or alpha diversity, when measured using the Shannon index (Welch’s *t*-test, *p* = 0.09, confidence interval, -0.04070364 – 0.43717834, degrees of freedom = 7.3003), of longfin eels between sampling locations (Lake Te Anau and Mavora Lakes) (Supplementary Fig. S1). We were unable to statistically measure if host species influenced richness or alpha diversity, measured by the Shannon index, as there were only two pooled samples of shortfin eels (Supplementary Fig. S1). Nevertheless, it appeared that shortfin eels had a higher overall richness and alpha diversity, measured by the Shannon index, compared to longfin eels (Supplementary Fig. S1). We were also unable to statistically test if the life stage of shortfin eels affected virome composition due to the small sample size.

## Discussion

We investigated the viromes of longfin and shortfin eels caught in three locations across the South Island of New Zealand, and in doing so, identified putative viruses belonging to eight different viral families, significantly enhancing our understanding of eel virus diversity in New Zealand. This study also expanded our understanding of the host range of these viruses. Notably, eight of the nine viral sequences identified here represented putative novel viruses, further highlighting the vast potential to discover new viruses within fishes [5, 69]. Fish viruses have historically been understudied, particularly given that fishes account for greater than 50% of the total vertebrate diversity [70]. Like in previous work [5, 69], the eel viruses identified here clustered with fish viruses, indicating long-term viral-host co-evolution among this class, at least on a broad scale.

Flaviviruses (in particular, hepaciviruses) were highly prevalent, with three new putative viruses identified. All longfin eel flaviviruses were genetically homogenous, with >90% amino acid sequence identity, when comparing across the conserved polymerase region, and formed a sister clade to shortfin eel flaviviruses, which is perhaps indicative of cross-species viral transmission or viral codivergence, although a broader sampling of eels of the family Anguillidae is necessary to confirm such patterns. Hepaciviruses, once thought to only infect mammals, have a broad host range and are typically associated with liver disease in humans [71]. Despite this, as our knowledge of hepacivirus diversity has expanded, disease-causing viruses are becoming less frequently detected [72–74]. The discovery of hepaciviruses in New Zealand’s eels further expands our understanding of the host range and genetic diversity of these viruses.

Viruses such as Eel virus European, Eel virus European X, and Anguillid herpesvirus 1 have been detected in wild and farmed eels across the world and cause severe hemorrhagic disease, resulting in significant mortality [37, 38]. While viruses of the families *Birnaviridae* and *Alloherpesviridae* were not found in this study of New Zealand’s eel viruses, we identified a rhabdovirus in shortfin eels caught in Te Waihora/Lake Ellesmere. This virus was most closely related to Wuhan redfin culter dimarhabodovirus, which was identified previously in predatory carp from China, and clustered phylogenetically with Porure dimarhabdovirus 1 identified in New Zealand common smelt (*Retropinna retropinna*). Based on their phylogenetic position, these viruses fell within the subfamily *Alpharhabdovirinae*, alongside Eel virus European X. While there is no evidence that shortfin eel rhabdovirus, which was identified in seemingly healthy shortfin eels, causes disease in these hosts, it is useful to understand the baseline diversity and abundance of these viruses to better identify and mitigate future disease outbreaks.

We have further expanded the known host range of members of the newly created viral family *Nanghoshaviridae*, identifying longfin eel nanghoshavirus in samples from both Lake Te Anau and Mavora Lakes. Currently, this family only includes shortfin eel nanghoshaviruses 1 and 2 sampled from a remote New Zealand Chatham Island lake [35], Nanhai ghost shark arterivirus identified in ghost sharks in China [5], and Neolamprologus nanghoshavirus identified in a species of cichlid fish from Tanzania [75]. Very little is known about the members of the family *Nanghoshaviridae*, which were only formally classified in 2019. This family belongs to the suborder *Nanidovirineae* in the order *Nidovirales* [76]. It should be noted that none of the previously discovered nanghoshaviruses are known to cause disease, and all of the nanghoshaviruses discovered so far have also been identified in fish [35, 75, 76], indicating a possible marine origin of nanghoshaviruses, and potentially of the order *Nidovirales* in general [77].

The shortfin eels examined here were primarily used in a larger experiment not associated with this study before tissues were sampled for RNA sequencing. During this time, there was the potential for viruses to be transmitted between co-housed silvering and yellow shortfin eels, particularly since the experimental manipulation conceivably induced stress in the animals. Stressed fish are known to release cortisol, suppressing the inflammatory response and thus increasing susceptibility to viral infection [78]. Additionally, stress has also been associated with lowering antibody responses and impairing antiviral innate immune responses, further increasing susceptibility to disease [78, 79]. Nevertheless, aside from flavivirus sequences that were identified in both silvering and yellow shortfin eels, which may indicate viral codivergence rather than cross-species virus transmission, there is no other evidence of viral transfer between the two shortfin eel samples. Indeed, the viral richness was similar between longfin and shortfin eels, indicating that that the differences in sampling and handling strategy likely had minimal effect on the virome composition overall.

Virome composition is often driven by host specificity as well as environmental factors [35, 69]. For example, the diversity of viruses in Chatham Island fishes was found to be significantly host-specific [35]. Comparatively, analysis of the Pacific Ocean Virome dataset identified environmental factors, including geographic region, depth, and proximity to the shore, that significantly influence virome composition [80]. Similarly, we found that both host species specificity and location (of longfin eels) appeared to be important for shaping virome composition, although this requires further scrutiny because of the small sample sizes involved. Consequently, further sampling is required to obtain a better understanding of the extent to which these factors influence virome composition. However, it is particularly interesting that the location of longfin eels in this study significantly influenced virome composition, given that elvers from the Manapōuri Lake Control Structure are translocated manually to Lake Te Anau [42, 45]. Nevertheless, given that longfin eels can spend 20-90 years in their freshwater environments before migrating to the Pacific Ocean [81, 82], it is perhaps unsurprising that viruses in these hosts evolve location-specific differences, even with species translocations. It will be valuable in the future to further explore location-specific differences between the lakes and determine whether characteristics such as lake temperature, salinity, depth, and diet also influence eel virome composition.

We have expanded our knowledge of the viruses in New Zealand’s longfin and shortfin eels. Both host specificity and geography seemingly contribute to virome composition, highlighting the complex interaction between viruses, their hosts, and their ecosystems. These insights help broaden our understanding of aquatic host viromes, emphasising the importance of such studies to reveal the viromes of healthy species. This information can be used in the future alongside other more-extensive pathological studies to form a baseline to compare changes in virus diversity during disease outbreaks or translocations of species or to monitor the effect climate change has on virome composition over time.

### Supplementary Information

Below is the link to the electronic supplementary material.

Supplementary file1. Supplementary Table S1. Data surrounding eel library pooling and sampling site locations (XLSX 10 KB)

Supplementary file2. Supplementary Table S2. Data surrounding the viral contigs that were identified in this study and the top BLASTp results (XLSX 14 KB)

Supplementary file3. Supplementary Fig. S1 Richness (left) and Shannon index analysis (right) (a) of longfin eel viruses across longfin eel libraries in relation to location (Te Anau and Mavora). Significant differences in richness and Shannon index were measured using Welch’s t-test. Richness (left) and Shannon index analysis (right) (b) of eel viruses across eel libraries in relation to species (longfin eels and shortfin eels) (PDF 506 KB)

## Data Availability

The raw sequencing reads generated in this project are available in the Aotearoa Genomic Data Repository, DOI number 10.57748/7TDA-0G64, and the viral sequences have been submitted to GenBank under the accession numbers OR863200-OR863225 (Supplementary Table S2). Alignments and code for the statistical analysis can be found at https://github.com/stephwaller/NZ-Eel-Virome-Paper.git.
