# Supplementary figures and images for: Host and geography impact virus diversity in New Zealand’s longfin and shortfin eels
Source: Arch Virol. 2024 Mar 28;169(4):85. doi: 10.1007/s00705-024-06019-1 (PMC10978610; doi:10.1007/s00705-024-06019-1)

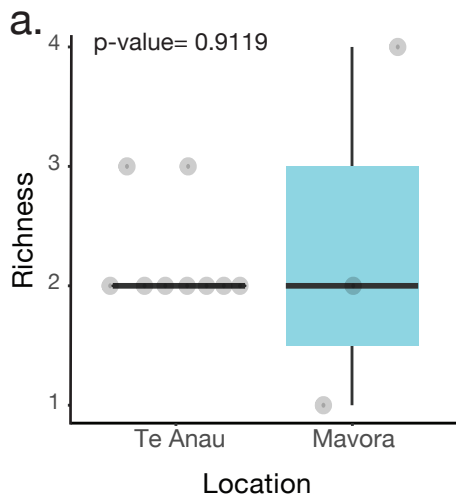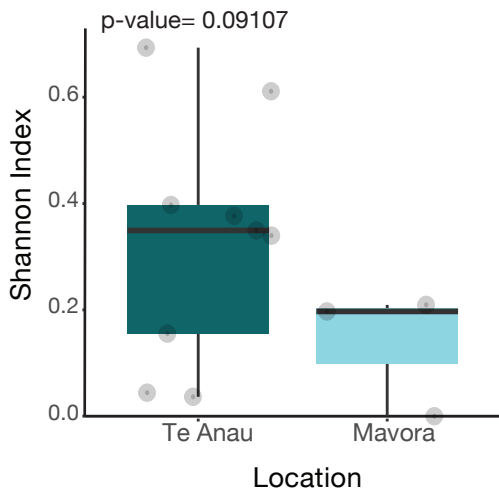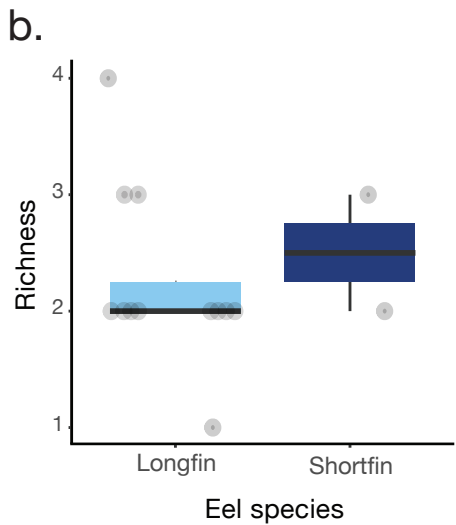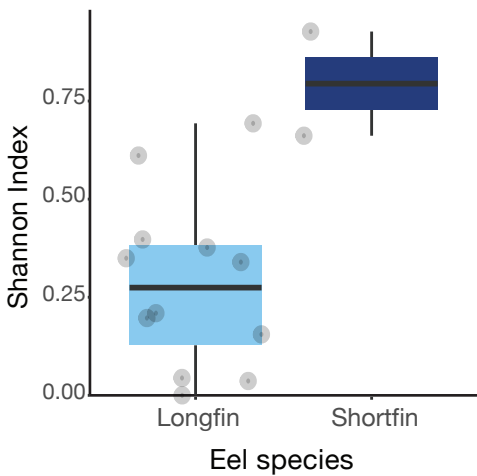

Supplement: Supplementary file 3 — Supplementary file3. Supplementary Fig. S1 Richness (left) and Shannon index analysis (right) (a) of longfin eel viruses across longfin eel libraries in relation to location (Te Anau and Mavora). Significant differences in richness and Shannon index were measured using Welch’s t-test. Richness (left) and Shannon index analysis (right) (b) of eel viruses across eel libraries in relation to species (longfin eels and shortfin eels) (PDF 506 KB) [file 705_2024_6019_MOESM3_ESM.pdf]
